# Aging, inflammation, stem cells, and bone healing

**DOI:** 10.1186/s13287-016-0300-9

**Published:** 2016-03-22

**Authors:** Emmanuel Gibon, Laura Lu, Stuart B. Goodman

**Affiliations:** Department of Orthopaedic Surgery, Stanford University, R116, 300 Pasteur Drive, Stanford, CA 94305 USA; Laboratoire de Biomécanique et Biomatériaux Ostéo-Articulaires - UMR CNRS 7052, Faculté de Médecine - Université Paris7, 10 avenue de Verdun, 75010 Paris, France; Department of Orthopaedic Surgery, Hopital Cochin, APHP, Université Paris5, 27 rue du Faubourg Saint-Jacques, 75014 Paris, France

## Abstract

Complex interactions among cells of the monocyte-macrophage-osteoclast lineage and the mesenchymal stem cell-osteoblast lineage play a major role in the pathophysiology of bone healing. Whereas the former lineage directs inflammatory events and bone resorption, the latter represents a source of cells for bone regeneration and immune modulation. Both of these lineages are affected by increasing age, which is associated with higher baseline levels of inflammatory mediators, and a significant reduction in osteogenic capabilities. Given the above, fracture healing, osteoporosis, and other related events in the elderly present numerous challenges, which potentially could be aided by new therapeutic approaches to modulate both inflammation and bone regeneration.

## Background

Most developed countries are facing an aging population. Currently, persons over 65 years of age represent 13 % of the American population [1], and this number is expected to grow as the “Baby Boomer” generation continues to age beyond 65 [2]. By 2030, they are projected to represent 16.9 % of the population; this segment will increase to 25.8 % by 2060 [3]. The changing demographics of the world’s population have wide-ranging implications that include a shift in medical needs.

Bone fractures are among the most common orthopedic problems that require medical intervention, particularly in the elderly. Almost half of fractures are related to osteoporosis, especially in individuals over the age of 55 [4]. Beyond the impact on the health and quality of life of individual patients, fractures are expensive and present a multi-billion-dollar cost to society because of direct and indirect costs [4]. With an increasingly aging population, a better understanding of how bone repair changes with age is critical in developing and optimizing effective therapeutic treatments.

Bone healing is a complex process. After bone injury, a stage of inflammation is necessary for progression to healing. In vivo studies have shown early secretion of pro-inflammatory factors such as interleukin (IL)-1 and IL-6, tumor necrosis factor-alpha (TNFα), macrophage colony-stimulating factor (M-CSF), and inducible nitric oxide synthase (iNOS) [5]. In a study of double *TNFα* gene knockout mice (*p55*^−/−^/*p75*^−/−^), Gerstenfeld et al. [6] showed that pro-inflammatory signals are required for proper bone repair, as these mice failed to initiate intramembranous bone formation and had markedly reduced expression of type 1 collagen and osteocalcin mRNA. Moreover, Xing et al. [7], using *CCR2*^−/−^ mice, have shown that inflammation is critical to bone healing; when the CCR2-monocyte chemotactic protein-1 (CCR2-MCP-1) chemokine-receptor axis was interfered with, inflammation and bone healing were impaired.

Bone marrow macrophages (also called osteal macrophages) are also important for the repair of bone by coordinating the crosstalk between osteoclasts and osteoblasts [8]. Furthermore, using the macrophage Fas-induced apoptosis (MAFIA) transgenic model, Cho et al. [9] showed that osteal macrophages mediated parathyroid hormone-dependent bone regeneration. Other studies also reported the important role of osteal macrophages in the processes of bone healing [10–12].

Beyond pro-inflammatory signals, macrophages also secrete many growth factors and chemokines that are critical during the inflammatory phase of bone healing [6, 13]. These growth factors include transforming growth factor-beta (TGFβ), insulin-like growth factor (IGF), fibroblast growth factor (FGF), and platelet-derived growth factor (PDGF). Macrophages also secrete chemokines, such as MCP-1 and monocyte inflammatory protein 1 alpha (MIP-1α), that are essential for mesenchymal stem cell (MSC) homing and migration to the injured site [14].

In addition to macrophages, MSCs are critical for bone regeneration. MSCs are multipotent and can differentiate into many cell types, including chondrocytes and osteoblasts for endochondral and intramembranous ossification, respectively [14]. A key step in bone healing is the localization of MSCs to the site of injury. For example, in a parabiosis model, Shinoara et al. [15] demonstrated that the stromal cell-derived factor 1/CXCR4 (SDF-1/CXCR4) ligand-receptor axis is critical for the homing of progenitor cells that participate in fracture healing. Similarly, Kitaori et al. [16] used an exchanging-graft and autograft mouse model to show that SDF-1^+/−^ and CXCR4^+/−^ are important to the recruitment of MSCs during skeletal repair. Many other studies have confirmed the beneficial role of MSCs in bone regeneration [17, 18]. Nevertheless, the origin of the MSCs that are directly involved in fracture healing is controversial. Colnot et al. [19] showed that periosteum and endosteum are primary sources of MSCs for fracture repair. Similarly, using a parabiotic mouse model, Kumagai et al. [20] showed little to no contribution of circulating cells to direct repair of the injured bone. At a minimum, systemically migrated MSCs and osteoprogenitors are thought to play an important paracrine role, modulating both inflammation and subsequent bone repair.

**Fig. 1:**
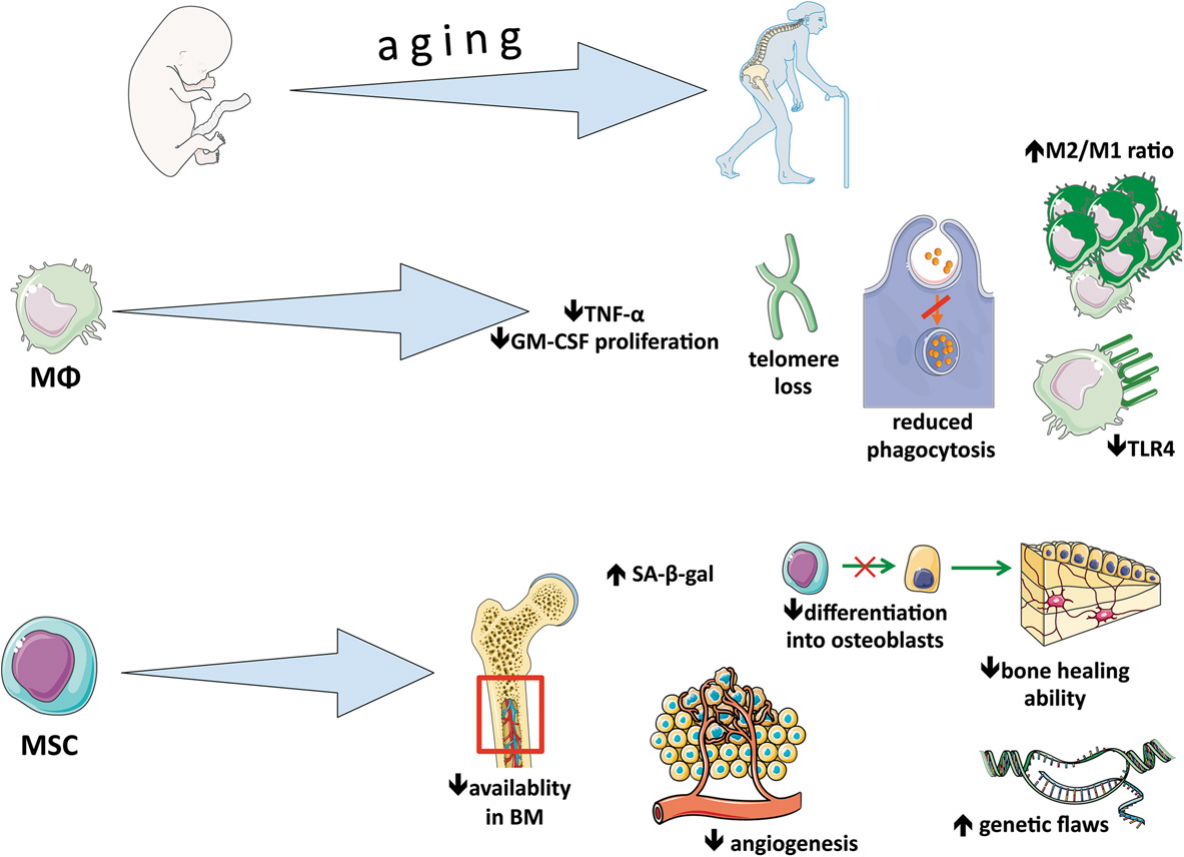
The effect of aging on mesenchymal stem cells and macrophages. ↑ increase, ↓ decrease, *BM* bone marrow, *GM-CSF* granulocyte macrophage colony-stimulating factor, *MΦ* macrophage, *MSC* mesenchymal stem cell, *SA-β-gal* senescence-associated β-galactosidase, *TLR4* Toll-like receptor 4, *TNF-α* tumor necrosis factor-alpha

Given the above, there appears to be a deficiency in our understanding of the interactions between macrophages and MSCs in bone healing, especially in the elderly population. Specifically, aging may alter these interactions and thereby play an important role in the elderly patient’s ability for regeneration of musculoskeletal tissues. This review will address the effect of aging on both macrophages and MSCs as it relates to bone healing. Figure 1 summarizes the effect of aging on MSCs and macrophages.

### The concept of macrophage polarization

Bone injury leads to the production of pro-inflammatory cytokines and chemokines and to systemic recruitment of macrophage precursors to the injury site [21]. As such, it is important to understand the different macrophage populations that play a role in bone repair. Though they exist within a spectrum, macrophages can be broadly described as uncommitted M0, pro-inflammatory M1, and anti-inflammatory M2 populations [22]. Mantovani et al. [23] have shown that these designations are similar in humans and mice. M0 macrophages can be polarized to pro-inflammatory M1 macrophages by interferon-gamma (IFNγ) and lipopolysaccharide (LPS) via Toll-like receptors (TLRs) like TLR-4, whereas both M0 and M1 macrophages can be polarized to an anti-inflammatory M2 phenotype by exposure to IL-4 [24, 25]. M1 macrophages are characterized by a cytokine release profile of TNFα, IL-6, IL-1, IL-12, IL-23, Oncostatin M (OSM), and type 1 IFN with increased expression of iNOS, CCR7, and HLA-DR [26, 27]. Alternatively, the M2 cytokine release profile includes IL-4, IL-10, IL-13, and IL-1ra and increased expression of CD206, Ym1 (eosinophil chemotactic factor), CD163, CCL1, CCL18, FIZZ1, Arginase 1, and chitotriosidase [28, 29]. In actuality, both in humans and mice, there probably exists a spectrum of polarization phenotypes, with a general preponderance of pro- versus anti-inflammatory properties. With these multiple phenotypes, macrophages play several roles within the bone-healing process, depending on their polarization status and environmental cues. For instance, in humans, retrieved periprosthetic tissues revised because of loosening and osteolysis demonstrated increased M1/M2 macrophage ratios [25].

## Aging and macrophages

Macrophages are essential components of the innate and adaptive immune systems, in the maintenance of physiological homeostasis, and in bone remodeling [11]. As these cells play an important role in a wide variety of processes, a clear knowledge of how macrophages function and how they change with age is crucial for understanding both healthy and pathological states.

### Intrinsic changes with aging

Although it is apparent that macrophages have altered activities with age, it is unclear as to what these specific changes entail and the mechanisms that drive such changes in musculoskeletal tissues. Several studies point to intrinsic factors that alter macrophage polarization, function, and survival. Wang et al. [30] found that aged murine muscle had higher levels of M2a polarized macrophages, muscle fibrosis, and collagen accumulation. The increased frequency of M2a macrophages and fibrosis was attributable to the aging of myeloid lineage cells, as demonstrated by rescue of aged muscle with infusion of young bone marrow cells [30]. Interestingly, inducing muscle-specific neuronal nitric oxide synthase (nNOS) was sufficient to prevent increased M2a frequency and arginase-1-dependent fibrosis [30]. Shortening of telomeres in aged macrophages also contributes to macrophage susceptibility to oxidative stress and reduced granulocyte macrophage colony-stimulating factor (GM-CSF)-dependent proliferation [31]. These cellular defects were found in aged and telomerase knockout (*Terc*^−/−^) mice; Sebastian et al. concluded that telomere loss caused reduced STAT5a oxidation and phosphorylation and ultimately impairment of GM-CSF-dependent macrophage proliferation [31]. Similarly, increased levels of S-endoglin, a transmembrane glycoprotein associated with inflammatory processes, were associated with decreased macrophage proliferation, decreased survival response to GM-CSF, increased oxidative stress, and skewed myeloid cell polarization toward an M2 phenotype [32]. Chitotrioside, a marker for chronically activated macrophages and inflammation, has also been shown to be elevated in older humans [33]. Herrero et al. demonstrated that, at the genomic level, aged macrophages have decreased DNA-binding activity in the promoter region of the *IAβ* gene, resulting in decreased expression of major histocompatibility complex (MHC) class II molecules [34]. As evidenced by the changes described above, aging alters many aspects of macrophage survival and function.

### Aging microenvironment

In addition to intrinsic changes of aging, macrophages are modulated by their aging microenvironment and a poorly described number of external factors. When challenging young macrophages with aged serum, Gomez et al. found reduced macrophage secretion of TNFα and increased basal levels of IL-6 [35]. Though the group did not specifically identify the factors contributing to these observations, they concluded that, owing to heat resistance of the effect, the stimulatory factor(s) for increased IL-6 production was not a protein [35]. In a study comparing phagocytosis by young and aged peritoneal macrophages and bone marrow-derived macrophages (BMDMs), Linehan et al. demonstrated that older peritoneal macrophages have significantly impaired phagocytosis compared with younger macrophages; however, there was no evident defect in phagocytosis for aged BMDM [36]. Moreover, they found that injection of young peritoneal macrophages into the peritoneal cavities of aged mice led to impaired phagocytosis and increased levels of T and B cells [36]. Barrett et al. found that glial cells exposed to conditioned media from aged BMDMs challenged with IFNγ or LPS had increased expression of pro-inflammatory mediators [37]. Such a pro-inflammatory environment could further mediate increased inflammation by infiltrating macrophages and thus contribute to a cascade of cellular damage [37]. Together, these findings suggest a profound influence of the aging microenvironment on macrophage function.

### Inflamm-aging

Aging is also associated with elevated levels of secreted inflammatory cytokines beyond the previously described functional and environmental changes [38]. Much of the literature describes aged macrophage hypersensitivity and increased responsiveness to inflammatory signals. For example, when aged BMDMs are challenged with IFNγ or LPS, they increase their expression of arginase and secrete characteristic pro-inflammatory M1 and Th1 cytokines, such as TNFα, NOS2, IL-1β, and IFNγ [30, 37, 39]. Moreover, aged macrophages increase their surface density of TLR4, the receptor for LPS, permitting a faster and enhanced inflammatory response [40]. Similarly, Smallwood et al. found that aged macrophages have increased nitric oxide production under resting conditions as well as enhanced bactericidal activity against *Salmonella* [41]. These findings suggest that aged macrophages remain in a pre-activated resting state that enhances their response to exposure of pro-inflammatory stimuli [41]. However, with increased production of reactive oxygen species, aged macrophages are susceptible to oxidative damage [41]. Although there is increased responsiveness to pro-inflammatory signals, aged macrophages also have impaired function with reduced phagocytic activity, reduced nitrite burst capacity, and reduced autophagy [38].

Recently, the phenomenon of “inflamm-aging” has been challenged by several studies that have shown decreased macrophage responsiveness to inflammatory signals. Some studies have shown that aged macrophages are less responsive to IFNγ and LPS as evident by decreased macrophage-mediated tumoricidal activity and reduced secretion of TNFα, IL-1β, IL-6, iNOS, and IFNγ [42–44]. Though the mechanisms for these changes are still unclear, it has been shown that age-associated decrease in IFNγ responsiveness is at least partially mediated by the lack of tyrosine phosphorylation of mitogen-activated protein kinase (MAPK) [45]. Similarly, aged mice highly express miR-146a, a microRNA that negatively regulates IL-1β and IL-6 via LPS and the nuclear factor kappa-light-chain-enhancer of activated B cells (NFκB) pathways [43]. These differing findings suggest that aged macrophages can be modulated under various conditions and are likely part of a more dynamic interplay among intrinsic aging mechanisms, the microenvironment, and different populations of surrounding cells.

### Broader implications

Given current knowledge, it is apparent that aging-associated changes in the macrophage population are normal events but can also be potential sources for pathological states. As such, modulation of macrophages can provide an avenue for future therapeutics. For example, Slade Shantz et al. demonstrated that blocking macrophage activity by using PLX3397, a drug that blocks the kinase domain of CSF-1R, can accelerate bone callus maturation and subsequent bone formation, illustrating a potential means of enhancing fracture healing and preventing nonunion in the elderly [46]. Several studies showed that aging affects fracture healing in animal models. Histing et al. [47] compared fracture healing in both young and aged senescence-accelerated mice (SAMP6) and senescence-resistance mice (SAMR1). Fracture healing was delayed in aged SAMP6 mice compared with aged SAMR1 mice. The authors concluded that increased osteoclast activity in aged SAMP6 mice was responsible for the difference. However, Egermann et al. [48], using the same model, did not find any differences. Interestingly, using a chimeric model, Xing et al. [49] showed that aged mice receiving juvenile bone marrow cells could accelerate their age-related delay in fracture healing. A decrease in cyclooxygenase 2 (COX-2) expression in the early inflammatory phase of bone repair resulting in delayed remodeling in aged mice was observed by Naik et al. [50]. Lu et al. [51] found a decreased number of chondrocytes expressing collagen II and osteoblasts expressing osteocalcin in middle-aged and elderly mice, compared with younger mice.

With regard to therapy, in order to target age-related inflammation, clinicians have used estrogen to treat a variety of inflammation-mediated conditions, including traumatic injuries [52]. With a better understanding of how macrophages change with age and mediate different disease states, new therapeutics that specifically target these aspects of macrophage function can be developed.

## Aging and mesenchymal stem cells

The use of MSCs and MSC-derived osteoprogenitors in orthopedic surgery is gaining more widespread acceptance. Hernigou et al. pioneered the use of MSC-derived osteoprogenitors to treat osteonecrosis of the hip and other conditions involving bone healing [53]. A recent study extended the use of harvested osteoprogenitors to treat secondary osteonecrosis of the knee with promising results [54]. However, the management and use of MSCs are nuanced, and Prockop [55] has shown that the microenvironment into which MSCs are injected is critical and involves inter-cellular communication via soluble factors and complex cellular interactions. The effect of aging on MSCs is highly relevant, as cell-based therapies for both regeneration and immune modulation are developing rapidly.

### Abundance and growth

As the skeleton ages, the quantity of MSCs in the bone marrow decreases. Quarto et al. [56] compared the number of bone progenitor cells in adult and aged rats and found a significantly decreased number of bone progenitors in the bone marrow with aging. However, Chen [57] observed that the total number of MSCs harvested from mice was significantly higher in older mice (by approximately 20 %), but the older mice failed to produce as many osteoprogenitor cells compared with younger mice. Using specimens harvested from the iliac crest in healthy patients aged 5 to 70 years, Shigeno and Ashton [58] showed a significant decrease in both the number of precursor cells and degree of proliferation starting in the second and third decades of life. Likewise, Muschler et al. [59] investigated the dependence of nucleated cell and osteoblastic progenitor numbers in bone marrow aspirates on the basis of the age and gender of the patient. Expectedly, the total number of nucleated cells decreased with age regardless of gender, but surprisingly the number of osteoblastic progenitors did not decrease significantly for men whereas it did for women. Moreover, Stolzing et al. [60] also found a decrease in the number and proliferative capacity of MSCs harvested in older humans. Taken together, these data indicate that aging decreases the availability and growth potential of MSCs for bone formation. Furthermore, these changes may be dependent on the sex of the host.

### Differentiation, effectiveness, and intrinsic changes

The potential for differentiation of MSCs according to age is controversial, but most of the studies have shown a decrease in their capacity to undergo osteogenic differentiation. Baxter et al. [61] harvested human MSCs (hMSCs) from donors aged 0 to 18 (hMSCs_0-18_) and 59 to 75 (hMSCs_59–75_). The proliferative capacity and number of colony-forming-unit alkaline phosphatase-positive (CFU-ALP^+^) cells were decreased in hMSCs_59–75_. The authors also measured the mean telomere restriction fragment (mTRF), which can be used to estimate the remaining replicative capacity of a cell population. They found that mTRF in hMSCs_0–18_ was significantly longer than in hMSCs_59–75_. In another study, Zhou et al. [62] reported on cultured hMSCs from donors aged 17 to 90. Aged hMSCs showed increased numbers of senescence-associated β-galactosidase (SA-β-gal)-positive cells, apoptotic cells, a decreased proliferation rate, and ALP^+^ cells. Aged hMSCs also experienced genetic flaws with overexpression of *p53* and its target *p21* and *BAX* (apoptosis regulator) genes (apoptotic pathway). Kuehn [63] also reported major genetic flaws such as chromosomal rearrangement or overexpression of the *MYC* oncogene. Similarly, D’Ippolito et al. [64] found significantly fewer CFU-ALP^+^ cells in cultured MSCs from vertebral bone marrow from older donors. A potential explanation of the failure of aged MSCs to differentiate may be due to shortening of telomere length. To test this hypothesis, Liu et al. compared telomerase knockout MSCs (mTR^−/−^MSCs) to wild-type MSCs (WT-MSCs) [65]. Their results showed a complete failure of mTR^−/−^MSCs to differentiate into chondrocytes. Moreover, mTR^−/−^MSCs experienced early morphologic alterations. Pignolo et al. [66] also validated this hypothesis by using a mouse model of Werner syndrome (premature aging). The role of cell cycle regulators has been shown to be critical for the regulation of cellular senescence. Among the cell cycle regulators, p16^INK4A^, which interferes with CDK4 and CDK6 cell cycle kinases, was found to be overexpressed in aged hMSCs [67].

Wound healing is also affected by senescence. Choudhery et al. [68], in an in vitro study, showed decreased wound-healing abilities with murine MSCs (mMSCs) harvested from aged mice. Interestingly, they also found a downregulation of vascular endothelial growth factor (VEGF), stromal cell-derived factor 1 chemokine (SDF-1), and protein kinase B (which is known to inhibit apoptosis) expression in aged mMSCs. Angiogenic potential was also dramatically decreased in aged mMSCs. The potential for regeneration of muscle by MSCs is also profoundly affected by senescence. Resident muscle MSCs, also known as satellite cells, lose their self-renewal abilities via alterations in FGF receptor 1 and p38αβ MAPK signaling, as shown by Bernet et al. [69].

## Conclusions

With aging, the proliferative and functional abilities of macrophages and MSCs are impaired because of a combination of intrinsic and environmental factors. As proper bone healing requires an inflammatory phase, the increased survival of anti-inflammatory M2 macrophages and reduced secretion of pro-inflammatory factors with age may jeopardize timely bone regeneration. At the same time, aging negatively impacts MSC proliferation and differentiation, further impeding the bone-healing process. It would appear that, taken together, both macrophages and MSCs, cells critical for regeneration of musculoskeletal tissues, are adversely affected by aging. This scenario provides new opportunities for modulation of cellular events in order to optimize the healing of mesenchymally derived tissues, including bone.

## Abbreviations

ALP: Alkaline phosphatase
BMDM: Bone marrow-derived macrophage
CCL1: Chemokine (C-C motif) ligand 1
CCL18: Chemokine (C-C motif) ligand 18
CCR2: C-C chemokine receptor type 2
CCR7: C-C chemokine receptor type 7
CD163: Cluster of differentiation 163
CD206: Cluster of differentiation 206
CDK: Cyclin-dependent kinase
CFU-ALP^+^: Colony-forming-unit alkaline phosphatase-positive
CSF-1R: Colony-stimulating factor 1 receptor
CXCR4: C-X-C chemokine receptor type 4
FGF: Fibroblast growth factor
FIZZ1: Found in inflammatory zone protein
GM-CSF: Granulocyte macrophage colony-stimulating factor
HLA-DR: Human leukocyte antigen-antigen D related
hMSC: Human mesenchymal stem cell
IFNγ: Interferon gamma
IL: Interleukin
iNOS: Inducible nitric oxide synthase
LPS: Lipopolysaccharide
MAPK: Mitogen-activated protein kinase
MCP-1: Monocyte chemotactic protein-1
miR-146a: Microrna-146a
mMSC: Murine mesenchymal stem cell
MSC: Mesenchymal stem cell
mTRF: Mean telomere restriction fragment
MYC: Myelocytomatosis
NOS2: Nitric oxide synthase 2
SAMP6: Senescence-accelerated mice
SAMR1: Senescence-resistance mice
SDF-1: Stromal cell-derived factor 1
STAT5a: Signal transducer and activator of transcription 5A
TLR: Toll-like receptor
TNFα: Tumor necrosis factor-alpha
WT-MSC: Wild-type mesenchymal stem cell
